# The effects of HIV self-testing on the uptake of HIV testing and linkage to antiretroviral treatment among adults in Africa: a systematic review protocol

**DOI:** 10.1186/s13643-016-0230-8

**Published:** 2016-04-05

**Authors:** Bernard Njau, Damian J. Damian, Leila Abdullahi, Andrew Boulle, Catherine Mathews

**Affiliations:** School of Public Heath and Family Medicine, University of Cape Town, Cape Town, 7925 South Africa; Vaccines for Africa Initiative, Institute of Infectious Disease and Molecular Medicine, University of Cape Town, Cape Town, 7925 South Africa; Department of Clinical Laboratory Sciences, Division of Medical Microbiology, Cape Town, 7925 South Africa; Health Systems Research Unit, South African Medical Research Council, Cape Town, South Africa

**Keywords:** HIV self-testing, Uptake, Yield, Barriers, Facilitators, Social harms, Africa

## Abstract

**Background:**

HIV is still a global public health problem. More than 75 % of HIV-infected people are in Africa, and most of them are unaware of their HIV status, which is a barrier to accessing antiretroviral treatment. Our review aims, firstly, to determine whether HIV self-testing is an effective method to increase the uptake of testing, the yield of new HIV-positive diagnoses, and the linkage to antiretroviral treatment. Secondly, we aim to review the factors that facilitate or impede the uptake of HIV self-testing.

**Methods/design:**

Participants will be adults living in Africa. For the first aim, the intervention will be HIV self-testing either alone or in addition to HIV testing standard of care. The comparison will be HIV testing standard of care. The primary outcomes will be (i) uptake of HIV testing and (ii) yield of new HIV-positive diagnoses. The secondary outcomes will be (a) linkage to antiretroviral (ARV) treatment and (b) incidence of social harms. For the second aim, we will review barriers and facilitators to the uptake of self-testing. We will search PubMed, Cochrane Central Register of Controlled Trials, Scopus, Web of Science, WHOLIS, Africa Wide, and CINAHL for eligible studies from 1998, with no language limits. We will check reference lists of included studies for other eligible reports. Eligible studies will include experimental and observational studies. Two authors will independently screen the search output, select studies, and extract data, resolving discrepancies by consensus and discussion. Two authors will use Cochrane risk of bias tools for experimental studies, the Newcastle-Ottawa Quality Assessment Scale for observational studies, and the Critical Appraisal Skills Programme (CASP) quality assessment tool for qualitative studies.

**Discussion:**

Innovative and cost-effective community-based HIV testing strategies, such as self-testing, will contribute to universal coverage of HIV testing in Africa. The findings from this systematic review will guide development of self-testing policy in African countries.

**Systematic review registration:**

PROSPERO CRD42015023935

**Electronic supplementary material:**

The online version of this article (doi:10.1186/s13643-016-0230-8) contains supplementary material, which is available to authorized users.

## Background

Globally, an estimated 35.0 million people are living with HIV, with more than 2.1 million (1.9 million–2.4 million) new HIV infections and 1.5 million deaths in 2013 [1]. Africa remains the most affected region, contributing more than two thirds of the global burden of HIV [2]. The UNAIDs/WHO has set a “90-90-90” global target to be reached by 2020. The global target stipulates that 90 % of adults will know their HIV status, 90 % of HIV positive will receive sustained antiretroviral (ARV) treatment, and 90 % of those who are on ARV treatment will achieve viral load suppression by 2020 [3].

Despite substantial efforts to increase HIV testing, particularly in populations with generalized HIV epidemics, testing coverage in Africa is low. A recent review of Demographic and Health Surveys reported that across 29 Sub-Saharan Africa (SSA) countries, only 15 % of adults received HIV test result in the past 12 months [4]. The observed low coverage is a critical barrier to scaling up HIV prevention, care, and treatment interventions. In addition, evidence suggests that most HIV-positive people in Africa seek late entry to ARV treatment and the delay has adverse impact on treatment outcomes, including high mortality, avoidable morbidity, and transmission of the virus [5, 6].

Evidence shows that HIV self-testing (HIVST) has the potential to empower non-testers to know their HIV status by overcoming facility-based barriers to HIV testing [7]. However, HIV policy makers have reservations about the introduction of self-testing and raise concerns regarding HIVST. The concerns vary from lack of policies and regulatory systems, quality of HIVST kits, ethical and human rights issues, and knowledge gaps pertaining to HIVST [8–10].

Apart from general concerns described above, there are several barriers associated with uptake of HIVST. Perceived accuracy of self-testing kits is a major barrier to uptake of HIVST, with evidence of uncertainty on perceptions of the accuracy of self-test kits [11]. The invasive nature of a finger prick for obtaining a blood sample is another barrier, particularly for blood-based self-testing because some people fear needle pricks [12]. The cost of buying self-test kits is another barrier. Most consumers have to pay for the self-test kits, ranging from US$ 4.8 to US$ 40, which may not be affordable to most people in low- and middle-income countries (LMICs), including SSA [8, 13]. Finally, illiteracy is another barrier associated with uptake of HIVST. Evidence shows that illiterate participants are less likely to undertake self-testing compared to their literate counterparts, particularly in an unsupervised self-testing strategy [13]. The current evidence on barriers to uptake of HIVST is from studies conducted in high-income settings and only four studies from Africa.

Three systematic reviews provided evidence on self-testing, globally. The reviews focused on HIVST strategies in low- and high-risk populations and acceptability of HIVST in high- and low-income countries. Pai et al.’s review reported a high acceptability, ranging from 74 to 96 % in high- and low-income countries. In addition, the review observed a higher preference for self-testing compared to facility-based testing and oral-based rather than blood-based self-testing [14]. Krause et al.’s review found that 50 to 60 % of participants were first-time testers. In addition, the review provided evidence of high performance accuracy rates of 99 % among laypersons compared to health-care providers. The review concluded that HIVST has a potential to reach first-time testers with high performance accuracy rates among laypersons [15]. Suthar et al.’s review, which compared different community-based HIV testing approaches, reported participant’s high uptake of self-testing of 86.9 % compared with 62 % of a school-based HIV testing. The review concluded that community-based HIV approaches, including self-testing, have high rate of testing uptake [16]. The current reviews focused mainly on studies from high-income settings, with only four studies (Malawi *n* = 3; Kenya = 1), were from African countries. The four studies lack evidence on yield of new HIV-positive diagnoses, post-test linkage to ARV treatment, and incidence of social harms post-testing [17]. However, since the last reviews in 2014, five new African studies on self-testing have been published.

Of importance is the fact that apart from WHO recommendation for introduction of HIVST as a strategy to increase universal coverage of HIV testing and counseling (HTC) [18], most African countries lag behind in terms of integration of HIVST in their national HTC guidelines [9]. In addition, there is a concern that with HIV self-testing, linkage to treatment (i.e., HIV-positive clients receiving sustainable ARV treatment) might be compromised [19]. A key challenge with HIVST implementation is ensuring people who test positive are not alienated from health services where ARV treatment is provided, because of the privacy and lack of post-test counseling associated with self-testing [8]. In order to answer this pertinent question, we propose to conduct this review. The aim of this study is to review the existing evidence on the effects of HIVST on the uptake of testing, the yield of new HIV-positive diagnoses, linkage to ARV treatment, incidence of social harms, and the factors that facilitate and impede the uptake of HIVST among adults in Africa.

## Aims

The first aim is to evaluate the effectiveness of HIV self-testing interventions on (i) the uptake of HIV testing, (ii) the yield of new HIV-positive diagnoses, linkage to ARV treatment, and (iii) incidence of social harms among adults in Africa.

The second aim is to review the barriers and facilitators to the uptake of self-testing among adults in Africa.

## Methods

This review has been registered in the PROSPERO International Prospective Register of systematic reviews (http://www.crd.york.ac.uk/PROSPERO), registration number CRD42015023935 [20].

## Eligibility criteria

### Study designs

For the review of the intervention effects, experimental studies including randomized controlled trials (RCTs), controlled before/after studies, and interrupted time series studies will be considered for inclusion in this review. Experimental studies will assess the effects on uptake, yield, incidence of social harms, and linkage to ARV treatment. In addition, given that this is an emerging area of research, we will conduct secondary supplementary analysis of observational studies that assessed the outcomes of interest in this review. These studies will be included in a separate section of the review results.

For the review of barriers and facilitators, we will include predominantly observational studies which include data on the factors which facilitate or impede the uptake of self-testing. We will employ a broad definition of observational studies and include all studies that utilize qualitative methods for data collection (i.e., focus groups, in-depth interviews, observation, and review of documents) and analysis by the contextual attributes, such as factors associated with gender, culture, ethnicity, and geographical settings.

### Participants

The study participants are adults (males and females) from African countries.

### Intervention

The intervention of this study is HIV self-testing. HIV self-testing refers to a process whereby an individual, who is willing to know his/her HIV status, collects his/her own specimen, performs the HIV test, and interprets the results in private at home or in other settings.

For the review of the intervention effects, the intervention condition is HIV self-testing, either alone or in addition to the standard of care in HIV testing. The comparison condition is HIV testing standard of care. This might involve (1) provider-administered testing, (2) door-to-door testing, (3) mobile testing, (4) index testing, (5) work place testing, and (6) client-initiated testing or some combination of these approaches.

### Outcomes

For the review of the intervention effects, the primary outcomes are uptake of testing and yield of new HIV-positive diagnoses among adults in African countries. The primary outcomes are defined as:

- Uptake: the proportion of individuals who underwent HIV testing and received their test results over those who were offered HIV testing [21].
- Yield of new HIV-positive diagnoses: the proportion of individuals who were newly diagnosed with HIV positive over those who were offered HIV testing [22, 23].

The secondary outcomes are defined as:

- Linkage into ARV treatment: proportion of newly diagnosed HIV-positive adults who are enrolled in ARV treatment at any point in time post-testing over all newly diagnosed HIV-positive adult participants [24].

- Social harm: proportion of participants who report any episode of harm during or after HIV testing (e.g., intimate partner violence, coercive testing by a partner, or suicide) [25].

Editorials, reviews, perspectives, and studies not evaluating self-testing strategies (e.g., home-based testing) will be excluded. Studies, which will not clearly define the type of HIV testing strategies or include subjects below 18 years old will also be excluded. We will not consider studies that included health-care providers. Any disagreements in study inclusion/exclusion will be resolved at a meeting between reviewers. For the qualitative studies, we will collect additional information concerning (a) barriers to the uptake of self-testing, (b) facilitators to the uptake of self-testing, and (c) experiences of adults who had participated in self-testing in any African countries.

### Setting

We will include studies conducted in any country on the African continent.

## Information sources

### Electronic databases

We will conduct two separate search strategies for aims one and two. For the review of the intervention effects, a comprehensive search strategy will be developed to identify both published and unpublished articles with no language restrictions from 1998 to 31st December 2015. This search restriction is used because since 1998, we have seen the emergence of advance developments of rapid HIV diagnostic tests (RDTs) including self-testing [26]. The review will search for related studies in PubMed, the Cochrane Central Register of Controlled Trails (CENTRAL), the Cochrane Database of Systematic Reviews (CDSR), Databases of Abstracts of Reviews of Effectiveness (DARE), Social Sciences Citation Index, Web of Science, and African Index Medicus. In addition, we will search websites and databases for gray materials such as World Health Organization Library Information System (WHOLIS), WHO Global Index Medicus, the Joint United Nations Programme on HIV/AIDS (UNAIDS resource library), Alliance of Health Policy and Systems Research, and The World Bank. The search strategies for electronic databases will incorporate Medical Subject Headings (MeSH), free-text terms, and comprehensive African search filter that will be adapted to suit each individual database using applicable controlled vocabulary [27, 28]. We will also check reference lists of included studies for other eligible reports.

For the review of barriers and facilitators, we will search for related studies in CINAHL and MEDLINE electronic databases using guidelines developed by the Cochrane Qualitative Research Methods Groups for searching for qualitative evidence [29]. Previous qualitative work has demonstrated that CINAHL and MEDLINE are the most important resource for qualitative evidence [30]. In addition to the abovementioned databases, we will search other resources for related articles, contact experts in the field, gray literature, and scan reference lists of relevant studies.

### Search strategy

For the review of the intervention effects, we will use various MeSH and search terms such as “adult,” “HIV,” “Human Immunodeficiency Virus,” “AIDS,” “Acquired Immunodeficiency Syndrome,” “self testing,” “HIV self-testing,” “HIVST,” “testing,” “counseling,” “provider-administered testing,” “uptake,” “yield,” “prevalence,” “HIV positivity,” “linkage,” “care,” “treatment,” Africa,” and “Africa South of the Sahara”. Searches will combine with the names of each country in Eastern, Northern, and Southern Africa by using the Boolean operators “OR” or “AND” (Additional file 1).

For the review of barriers and facilitators, we will use search terms for Boolean search strategy such as “adult,” “HIV,” “Human Immunodeficiency Virus,” “AIDS,” “Acquired Immunodeficiency Syndrome,” “self testing,” “HIV self-testing,” “HIVST,” “barriers,” “facilitators,” “HIV self-testing experiences,” Africa,” and “Africa South of the Sahara”. We will use various combinations of these terms with the search engines.

## Study records

### Data management

All search results will be merged into reference management software EndNote, and duplicate records of the same report will be removed.

### Selection process

Full copies of articles identified by the search, and which meet the inclusion criteria, based on the title and abstract will be obtained for data synthesis. Firstly, two reviewers will independently apply the inclusion criteria to the results of the searches, based on the titles and abstracts alone.

### Data collection process

Two reviewers will extract data using a pre-designed data extraction forms and summarize the most important information from each study independently. A third reviewer will be consulted to resolve any differences of opinion between the two reviewers if they may arise. We will conduct a pilot trial of both data extraction forms to check its adequacy and make changes if necessary.

### Data items

Where possible, extracted data will include study details, setting of the study (e.g., city/country/or rural/urban or facility-based/community-based), year of publication—1998 to date, and type of HIV self-testing (e.g., supervised or unsupervised) (Additional files 2 and 3).

### Risk of bias in included studies

Two reviewers will code each included study using Cochrane risk of bias tools for RCT studies [31] and the Newcastle-Ottawa Quality Assessment Scale for observational studies [32]. This will be supplemented with the Effective Practice and Organisation of Care (EPOC) “Risk of bias” guidance to assess the risk of bias of non-randomized studies [33]. Studies will be assessed on sequence generation; allocation concealment; blinding of participants, personnel, and outcome assessors; incomplete outcome data; selective outcome reporting; and other sources of bias and rated as low risk/or adequate, high risk/or inadequate, and unclear [34] (Additional file 2).

We will use the Critical Appraisal Skills Programme (CASP) quality assessment tool to assess the methodological quality of the qualitative studies [35, 36]. This tool includes the following 14 questions: (1) Is this study qualitative research? (2) Are the research questions clearly stated? (3) Have ethical issues been taken into consideration? (4) Is the qualitative approach clearly justified? (5) Is the approach appropriate for the research question? (6) Is the study context clearly described? (7) Is the role of the researcher clearly described? (8) Is the sampling method clearly described? (9) Is the sampling strategy appropriate for the research question? (10) Is the method for data collection clearly described? (11) Is the data collection method appropriate for the research question? (12) Is the method of analysis clearly described? (13) Is the chosen analytical approach suitable for addressing the research question? and (14) Are the claims made supported by sufficient evidence? [36]. We will conduct a pilot trial on four included studies to assess the feasibility of the use of the tool and ensure integrity of the assessment. The quality assessment for risk of bias will be cross-checked by a third reviewer for discrepancies.

### Quantitative data analysis and synthesis

We will express the results of each study as risk ratios with corresponding 95 % confidence intervals. We will combine the estimates according to the study design; that is, we will pool estimates for each stratum (by design of the study). We will not combine data across different types of design. Random effects meta-analysis will be preferred due to anticipated heterogeneity in study results. The study will use the log relative risks for intervention studies using the generic inverse variance method in Cochrane Review Manager [37]. If we encounter variation in reported outcome measures between studies, median effect sizes will be presented (with a range).

### Dealing with missing data

The study will make efforts to contact corresponding authors to request for clarification of all relevant information in case of missing data. In case the corresponding author fails to respondent within a week of requesting for information, other author(s) will be contacted (copying the first author). A full description of missing data and drop-outs for each included study will be elaborated in the risk of bias table and discuss the extent to which the missing data could alter the results. The study will conduct sensitivity analyses to assess the effect of missing data on the primary meta-analyses. Ongoing projects will be classified as studies awaiting classification.

### Unit of analysis issues

In case of investigators report on cluster-randomized trial data as if randomization was performed at individual level rather than the cluster, a request to study authors for the intra-cluster correlation coefficient (ICC) will be submitted. In case of failing to obtain the information, the study will seek external ICC estimates from similar studies or available resources [38]. The study will use the established ICC to reanalyze the trial data to obtain approximate correct analyses according to description in the Cochrane Handbook for Systematic Reviews of Interventions [34]. A sensitivity analyses to assess the potential bias that may have occurred as a result of the inadequately controlled clustered trials will be performed. Further, the study will perform sensitivity analyses if the ICCs were obtained from external sources to assess the potential biasing effects of inadequately controlled cluster-randomized trials [39]_._

### Assessment of heterogeneity

For the review of the intervention effects, heterogeneity will be assessed by inspecting a forest plot initially and later through the Cochran’s chi-square test using a 10 % level of significance cut-off, and *I*^2^ statistic where values of 25, 50, and 75 % reflect low, medium, and high heterogeneity, respectively [34, 37]. For the review of barriers and facilitators, we will record differences across the studies with regard to settings, participants, barriers, and facilitators to the uptake of self-testing, for example, and these will inform the analyses.

## Sensitivity analysis

In case the identified studies are similar enough that it would be sensible to combine them in a meta-analysis, we will conduct sensitivity analyses to investigate the robustness of the results to risk of bias (i.e., omitting any studies with high risk of bias) and method of meta-analysis (i.e., random effects vs. fixed effect). We will conduct sensitivity analysis to (i) evaluate the effect of excluding studies unable to meet each quality criterion affect the overall estimate and (ii) evaluate the change in the results if only high-quality studies where included.

### Assessment of reporting biases

The study will employ strategies to search for and include relevant unpublished studies in order to reduce publication bias. These strategies will include searching the gray literature, including conference proceedings (e.g., 1st International symposium on self-testing for HIV and International AIDS Society) and prospective trial registration database to overcome time-lag bias. A funnel plot will be used to investigate the risk of publication bias by intervention type, provided 10 or more studies are included in the analysis for each intervention type. The funnel plot will be critically examined for asymmetry by both visually and use of formal tests.

### Grading the quality of evidence

The study will use the Grading of Recommendations Assessment, Development and Evaluation (GRADE) approach to assess the quality of evidence related to each of the primary and secondary outcomes for the review of the intervention effects. The GRADE approach results in an assessment of the quality of a body of evidence will be categorized as high, moderate, low, and very low [40]. High-quality evidence refers to “further research is very unlikely to change our confidence in the estimate of effect.” Moderate-quality evidence implies that “further research is likely to have an important impact on our confidence in the estimate of effect and may change the estimate.” Low-quality evidence means “further research is very likely to have an important impact on our confidence in the estimate of effect and is likely to change the effect.” Evidence is considered of very low quality if “we have very little confidence in the effect estimate.” Two authors (BN and DD) will independently assess the quality of evidence as implemented and described in the GRADE profiler (GRADEpro) software [41].

For the review of barriers and facilitators, we will assess the certainty of findings from qualitative studies using the *CerQual* (certainty of qualitative evidence) approach. Certainty refers to how likely it is that the review finding happens in a context of the included studies and could happen elsewhere. In this approach, assessment of certainty is based on two factors: the methodological quality of individual studies and the plausibility of each study finding. We will use an adaptation of the CASP tool for qualitative studies to assess the methodological quality of individual studies [36]. We will also assess the plausibility of each study finding by looking at the extent to which we are able to identify a clear pattern across the individual study data. This pattern could include, for example circumstances where findings are consistent across multiple contexts or where the review finding incorporates explanations for any variability across individual studies. As a final step, we will prepare summary of findings of the qualitative evidence synthesis. The summary tables will provide key findings, the certainty of evidence for each finding, and explanation of the assessment of the certainty of the qualitative evidence [36]. We will use three levels to indicate the certainty of the qualitative evidence—high, moderate, and low. High certainty will refer to findings drawn from generally well-conducted studies with high levels of plausibility. Moderate certainty will refer to findings drawn from studies with concerns related to either plausibility or methodological quality. Low certainty will refer to findings drawn from studies with concerns regarding both the methodological quality and plausibility of the finding.

### Qualitative data analysis and synthesis

For the review of barriers and facilitators, we will conduct the qualitative synthesis for types of facilitators and barriers related to uptake of testing. The aim of the synthesis is to enhance understanding of questions regarding “what works for whom and in what context” and to identify “barriers” and “facilitators” to the uptake of self-testing. The qualitative data analysis will base on thematic synthesis of qualitative research. Two authors will independently code key descriptive themes on types of facilitators and barriers, related with uptake of self-testing. We will discuss the resulting themes and sub-themes within the study team to examine their relationship to the review outcomes. The qualitative synthesis will then proceed by using the “descriptive themes” to develop “analytical themes,” which will be interpreted in reference to the review aims.

## Discussion

Achieving universal coverage of HIV testing for the general populations in Africa with scarce resources requires the implementation of innovative and cost-effective community-based HIV testing strategies, such as self-testing. The findings from this systematic review will inform on the knowledge gaps on the use of HIVST, yield and linkage to ARV treatment, incidence of social harms, and facilitators or barriers to uptake of HIVST among adults in Africa. We anticipate that our findings will guide the development of HIV self-testing policy, which is virtually non-existent at present in most African countries.

## Presenting and reporting of results

This protocol will follow the Preferred Reporting Items for Systematic Review and Meta-Analysis Protocols (Additional file 4) 2015 Statement [42]. We will present the results of this review according to the Preferred Reporting Items for Systematic Reviews and Meta-Analyses (PRISMA) guidelines. A PRISMA flow chart will be produced to ensure transparency of the process [43]. A table of all included studies will be included in the final review, and the reasons for exclusion of studies will be clearly documented. Where statistical pooling is not possible, we will present the findings in narrative form using tables and figures to aid in data presentation.

## Interpretation of findings

The results of the review will be discussed in the context of the quality of evidence, the limitations of the review, and the strengths of the findings, with emphasis on their implications for the current HIV self-testing practice and the potential for future research.

## Additional files

Additional file 1: Appendix 1: Describing details of search strategy. (PDF 74 kb) Additional file 2: Appendix 2: Systematic review data extraction form: Observational quantitative and qualitative studies. (PDF 159 kb) Additional file 3: DATA EXTRACTION FORM. (DOCX 115 kb) Additional file 4: PRISMA-P (Preferred Reporting Items for Systematic review and Meta-Analysis Protocols) 2015 checklist: recommended items to address in a systematic review protocol. (DOC 81 kb)

## Abbreviations

CASP: Critical Appraisal Skills Programme
CDSR: the Cochrane Database of Systematic Reviews
CENTRAL: the Cochrane Central Register of Controlled Trials
CerQual: certainty of qualitative evidence
EPOC: Effective Practice and Organisation of Care
GRADE: Grading of Recommendations Assessment, Development and Evaluation
GRADEpro: GRADE profiler
MeSH: Medical Subject Headings
PRISMA: Preferred Reporting Items for Systematic Reviews and Meta-Analyses
PRISMA-P: Preferred Reporting Items for Systematic Review and Meta-Analysis Protocols
RevMan: Review Manager
