# Supplementary material for: The effects of HIV self-testing on the uptake of HIV testing and linkage to antiretroviral treatment among adults in Africa: a systematic review protocol
Source: Syst Rev. 2016 Apr 5;5:52. doi: 10.1186/s13643-016-0230-8 (PMC4822257; doi:10.1186/s13643-016-0230-8)
Supplement: Additional file 1: — Appendix 1: Describing details of search strategy. (PDF 74 kb) [file 13643_2016_230_MOESM1_ESM.pdf]

**Appendix 1: Describing details of search strategy**		
Concept		Search terms
Population	adults living in Africa	“adult”; adult*;HIV;”Human Immunodeficiency Virus”; “AIDS”; Acquired Immunodeficiency syndrome*; africa; africa*
Intervention	HIV self-testing	“HIV Infections” OR “HIV”[MeSH] OR “hiv”[tiab] OR hiv-1*[tiab] OR hiv-2*[tiab] OR hiv1[tiab] OR hiv2[tiab] OR hiv infect*[tiab] OR human immunodeficiency virus[tiab] OR human immunedeficiency virus[tiab] OR human immuno-deficiency virus[tiab] OR human immune-deficiency virus[tiab] OR (human immun*[tiab] AND deficiency virus[tiab]) OR acquired immunodeficiency syndrome[tiab] OR acquired immunedeficiency syndrome[tiab] OR acquired immuno-deficiency syndrome[tiab] OR acquired immune-deficiency syndrome[tiab] OR (acquired immun*[tiab] AND deficiency syndrome[tiab]) OR "sexually transmitted diseases, Viral"[MeSH:noexp]) AND (randomized controlled trial [pt] OR controlled clinical trial [pt] OR randomized [tiab] OR placebo [tiab] OR drug therapy [sh] OR randomly [tiab] OR trial [tiab] OR groups [tiab]) AND self-testing; self-test* HIV self-testing; HIVST; “testing”;“counseling” NOT (animals [mh] NOT humans [mh]).
Comparison	HIV testing standard of care	“provider-administered testing”; provider-administered test*;” testing standard of care”
Outcome	Uptake, yield and linkage into HIV treatment ,social harm	“uptake”; “yield”, “HIV positivity”;”social harm”
Africa		Africa [MeSH Terms] OR Africa [All Fields] (‘‘Africa’’[MeSH] OR Africa*[tw] OR Algeria[tw] OR Angola[tw] OR Benin[tw] OR Botswana[tw] OR ‘‘Burkina Faso’’[tw] OR Burundi[tw] OR Cameroon[tw] OR ‘‘Canary Islands’’[tw] OR ‘‘Cape Verde’’[tw] OR ‘‘Central African Republic’’[tw] OR Chad[tw] OR Comoros[tw] OR Congo[tw] OR ‘‘Democratic Republic of Congo’’[tw] OR Djibouti[tw] OR Egypt[tw] OR ‘‘Equatorial Guinea’’[tw] OR Eritrea[tw] OR Ethiopia[tw] OR Gabon[tw] OR Gambia[tw] OR Ghana[tw] OR Guinea[tw] OR ‘‘GuineaBissau’’[tw] OR ‘‘Ivory Coast’’[tw] OR ‘‘Cote d’Ivoire’’[tw] OR Jamahiriya[tw] OR Jamahiriya[tw] OR Kenya[tw] OR Lesotho[tw] OR Liberia[tw] OR Libya[tw] OR Libya[tw] OR Madagascar[tw] OR Malawi[tw] OR Mali[tw] OR Mauritania[tw] OR Mauritius[tw] OR Mayotte[tw] OR Morocco[tw] OR Mozambique[tw] OR Mozambique[tw] OR Namibia[tw] OR Niger[tw] OR Nigeria[tw] OR Principe[tw] OR Reunion[tw] OR Rwanda[tw] OR ‘‘SaoTome’’[tw] OR Senegal[tw] OR Seychelles[tw] OR ‘‘SierraLeone’’[tw] OR Somalia[tw] OR ‘‘South Africa’’[tw] OR ‘‘St Helena’’[tw] OR Sudan[tw] OR

	Swaziland[tw] OR Tanzania[tw] OR Togo[tw] OR Tunisia[tw] OR Uganda[tw] OR ‘‘Western Sahara’’[tw] OR Zaire[tw] OR Zambia[tw] OR Zimbabwe[tw] OR ‘‘Central Africa’’[tw] OR ‘‘Central African’’[tw] OR ‘‘West Africa’’[tw] OR ‘‘West African’’[tw] OR ‘‘Western Africa’’[tw] OR ‘‘Western African’’[tw] OR ‘‘East Africa’’[tw] OR ‘‘East African’’[tw] OR ‘‘Eastern Africa’’[tw] OR “Eastern African”[tw] OR “Northern Africa” [tw] OR ‘‘Southern Africa’’[tw] OR ‘‘Southern African’’[tw] OR ‘‘sub Saharan Africa’’[tw] OR ‘‘sub Saharan African’’[tw] OR ‘‘sub-Saharan Africa’’[tw] OR ‘‘sub-Saharan African’’[tw]) NOT (‘‘guinea pig’’[tw] OR ‘‘guinea pigs’’[tw] OR ‘aspergillums Niger’’[tw])
