# Supplementary material for: The effects of HIV self-testing on the uptake of HIV testing and linkage to antiretroviral treatment among adults in Africa: a systematic review protocol
Source: Syst Rev. 2016 Apr 5;5:52. doi: 10.1186/s13643-016-0230-8 (PMC4822257; doi:10.1186/s13643-016-0230-8)
Supplement: Additional file 2: — Appendix 2: Systematic review data extraction form: Observational quantitative and qualitative studies. (PDF 159 kb) [file 13643_2016_230_MOESM2_ESM.pdf]

Appendix 2: Systematic review data extraction form: Observational quantitative and qualitative studies

Uptake and yield of HIV Self-testing in Africa				
Date form completed[dd/mm/yyyy]			[..../..../......]	
**A: Source**				
Review author ID				
Study ID (surname of first author and year of first full report of study was published e.g. Njau,2001)				
Title				
Authors				
Contact details				
Published	Yes		No	
If yes, please provide citation/year of publication/name of journal/language				
References of potentially eligible studies from the reference list				
**B: Eligibility**				
Confirm eligibility for review	Yes		No	
Insert inclusion criteria for eacg characteristic as defined in the protocal				
Characteristics	page	Figure	Table	other
Decision taken	INCLUDE		EXCLUDE	
If no, give reason(s) for exclusion				
**DO NOT PROCEED IF STUDY EXCLUDED FROM REVIEW**				
**C:Methods**				
Aim(s) of the study				
Unit of analysis		by individuals	groups	focus
Ethical approval obtained	Yes	No	Unclear	Not reported
	If yes, which institutional review board?(with registration number):			
Study design	Quantitative study characteristics (Y/N)		Qualitative study characteristics (Y/N)	
	If yes,proceed to **Section D**		If yes,procced to **Section H**	
**D: Characteristics of quantitative study**				
Type of study	Cross-sectional	Cohort studies	Case-control	
Start date				
End date				
Total study duration (from recruitment to last follow-up)				

Country					
Data Source	Medical records	Special surveys	Multiple source(s)	Surveillance	Registries
If other sources describe					
Inclusion criteria					
Exclusion criteria					
Setting					
Method of recuitment of participants (e.g.,phone,mail,clinic patients, door-to-door,etc.)					
Informed consent	Oral	Written	Not done	Done, but method not reported	Not reported
Diagnostic criteria (e.g., oral rapid HIV test)					
Defined by standard criteria(Yes/No)					
Paricipants					
Total number enrolled					
Withdrawals and loss-to-follow up	Yes		No		
If yes, give reasons for withdrawals/loss-to-follow up					
Age	Range				
	mean				
	Median				
Sex	Number or % of males				
	Number of % of females				
Outcome measures					
Uptake of HIV self-testing	Number or % of individuals who underwent HTC and received their test results of those who were eligible				
Yield of new diagnoses	Number or % of individuals who were newly-diagnosed HIV-positive of those who were eligible				
Prevalence of newly diagnosed HIV positive	Number or % of individuals who were newly diagnosed HIV-positive of those who underwent HTC of those who underwent HTC				
Linkage to care and treatment	Number or % individuals who were newly-diagnosed HIV-positive and visited a clinic at least once after diagnosis in a				

	specific time frames					
Any other relevant inclusion or exclusion criteria						
**E: Results**						
Sample size						
Measure of the outcome(s)	Crude measure	Adjusted measure	Other(s)			
If adjusted, what factors were adjusted for in this study	List:					
Reported measure of the outcomes (Yes/No)						
Missing participants	Number					
	Reason(s)					
If missing data to be reported from the author (any communication with the author)	Yes	No				
If yes, please specify						
**F: Risk of bias assessment**						
Does the study have a high risk of:						
Selection bias? (allocation concealment,allocation sequence)	Yes		No		Unclear	
Reporting bias?	Yes		No		Unclear	
Are study results valid?	Yes		No		Unclear	
**G Miscellaneous**						
Funding source	Name					
	A source of bias?	Yes	Probably	Probably not	No	
Key conclusions of the study authors						
Miscellaneous comments from the study authors						
References to other relevant studies						
Correspondence required						
Miscellaneous comments by the review authors						
**H:Qualitative study characteristics**						
**Characteristics**	**Description as stated in report/paper**	**Location in text or source**				
Overall aim/purpose						
Research/analytical question(s)						
Methodology						
Geographical setting						
Cultural setting/social context						
Participants						

Data collection method:						
Survey						
In-depth interviews						
Focus group discussions						
Group discussions						
Other(s)						
Outcome(s)						
Thematic analysis						
Overall perception of uptake of HIV self testing						
Checklist for qualitative studies						
	Description and assessment			Comments	Location in text/source	
Type of qualitative study	Participant observation					
	Open-ended interviews					
	Structured interviews					
	Others(specify):					
**Theoretical approach**	Appropriate		Inappropriate		Not sure	
**1. Is a qualitative approach appropriate?**						
For example						
(a) Does the research questin seek to understand process or structures ,or illuminate subjective experinces or meanings?						
(b) Could a quantitative approach better have addressed the research question?						
**2. Is the study clear in what it seeks to do?**						
	Clear		Unclear		Mixed	
For example						
(i)Is the purpose of the study discussed: aims/objectives/research(s)?						
(ii)Is there adequate/appropriate refrenece to the literature?						
(iii) Are underpinning values/assumptions/theory discussed?						
**Study design**						
**3.How defensible/ rigorous is the research design/ methodology?**						
	Defensible		Indefensible		Not sure	
For example						
(a) Is the design appropriate to the resarch question?						
(b) Is a rationale given for using a qualitative approach?						
(c) Are there clear accounts of the rationale/justification for the sampling,data collection and data analysis techniques used?						
(d) Is the selectin of cases/sampling startergy theoretically justified?						
Data collection	Appropriate		Inappropriate		Not sure/inadequately reported	
**4.How well was the data collection carried out?**						
For example						
(i) Are the data collection methods clearly described?						
(ii) Where the approprtaite data collected to address the research question(s)?						
(iii)Was the data collection and record keeping systematic?						
**Trustworthiness**	Clearly described		Unclear		Not described	
**5. Is the role of the researcher clearly described?**						
For example						
(a)Has the relationship between the researcher and participants been adequately considered?						

(b) Does the paper describe how the research was explained and presented to the participants?						
**6.Is the context clearly described?**						
	Clear		Unclear		Not sure	
For example						
(i) Are characteristics of participants and setting clearly defined?						
(ii) Were observations made in sufficient variaety of circumstances?						
(iii) Was context bias considered?						
**7. Were the methods reliable?**						
	Reliable		Unrealible		Not sure	
For example						
(a) Was data collected by more than one method?						
(b) Is there justification for triangualation,or not for triangulating?						
(c) Do the methods investigate what they claim to?						
**Analysis**						
**8. Is the data analysis sufficiently rigorous?**						
	Rigorous		Not rigorous enough		Not sure/not reported	
For example						
(i) Is the procedure explicit(i.e.,is it clear how data was analysed to arrive at the results?)						
(ii) How systematic is the analysis,is the procedure reliable/dependable?						
(iii) Is it clear how the themes and concepts were derived from the data?						
**9. Is the data'rich'?**	Rich		Poor		Not sure/not reported	
For example						
(a) How well are the contexts of the data described?						
(b) How well has the detail and depth been demonstrated?						
(c) Are responses compared and contrasted across groups/sites/settings?						
**10. Is the analysis reliable?**						
	Reliable		Unreliable		Not sure/not reported	
For example						
(i) Did more than 1 researcher code/derive themes from the transcripts/data?						
(ii) If so how were differences resolved?						
(iii) were negative/dicrepant results addressed or ignored?						
**11. Are the findings convicing?**						
	Convicing		Not convicing		Not sure	
For example						
(a) Are the findings clearly presented?						
(b) Are the findings internally coherent?						
(c) Are the extracts from the original data included?						
(d) Are the data appropriatelly referened?						
(e) Is the reporting clear and coherent?						
**12. Are the findings relevant to the aims of the study?**						
	Relevant		Irrelevant		Partially relevant	
**13. Conclusions**	adequate		Inadequate		Not sure	
For example						
(i) How are links between data, interpretation and conclusions?						
(ii) Are the conclusions plausible and coherent?						
(iii) Have alternative explainations been explored and discounted?						
(iv) Does this enhance understanding of the research topic?						
(v) Are the implications of the research clearly defined?						
Is there adequate discussion of any limitations encountered?						

14. How clear and coherent is the reporting of ethics?						
	Appropriate		Inappropriate		Not sure/not reported	
For example						
(a) Have ethical issues been taken into consideration?						
(b) Are they adequately discussed (e.g.,do they address consent and anoymity?)						
(c) Have the consequences of the research been considered(i.e., raising expectations,changing behaviour?)						
(d) Was the study approved by an ethics committee (i.e., is ethics approval number available?)						
Other relevant information						
Key conclusions from the authors						
Notes						
