# Supplementary material for: The effects of HIV self-testing on the uptake of HIV testing and linkage to antiretroviral treatment among adults in Africa: a systematic review protocol
Source: Syst Rev. 2016 Apr 5;5:52. doi: 10.1186/s13643-016-0230-8 (PMC4822257; doi:10.1186/s13643-016-0230-8)
Supplement: Additional file 3: — DATA EXTRACTION FORM. (DOCX 115 kb) [file 13643_2016_230_MOESM3_ESM.docx]

**DATA EXTRACTION FORM**

STUDY ID: ------------------------------- Date form completed: ________________

Reviewer’s initials

**Part 1: COVER SHEET**

Study Title:

Authors:

Journal:

Contact details:

Language:

Citation:

**Part 2: STUDY CHARACTERISTICS**

Publication Year:………………………..Country of Study:……………………..

**Eligibility: (use attached check list)** Confirm eligibility for review? Yes

No

Decision taken: Included Excluded Pending

If not included, give reasons why:

**DO NOT PROCEED IF STUDY EXCLUDED FROM REVIEW**

**Methods:**

**Aim(s) of the study:**

**Ethical approval obtained:** Yes No Unclear Not reported

Study design: **Study population:** **Total numbers:**

**Observational:** Adults

Cross-sectional Males

Cohort-study Females

Both

Case-controls

**Experimental:**

RCTs controlled before/after interrupted series

Other: ________________________

**Study period** (from recruitment to end of study)

Start date: **-------/----/-----** End date: -------/----/----

**Data Source: Study setting**

Medical records Urban

Special survey Rural

Multiple sources Facility-based

Surveillance Community-based

Registries **Type of intervention:**

Other:_________________ HIVST

**Testing Methods** Provider testing (PITC)

Oral-based testing Client-initiated (CITC)

Finger-stick testing Door-to-door

Other:__________________ Mobile testing

Supervised HIVST Index testing

Unsupervised HIVST Workplace testing

Other:____________________

**Age groups included (describe):**

Age: Range:

Mean (standard deviation):

Median (interquartile range):

**Outcome Measures: (reported in the paper?)**

Uptake of HIV testing and counselling Yes No

Yield of new diagnosis Yes No

Prevalence of HIV positive Yes No

Linkage to HIV prevention Yes No

Linkage to care Yes No

Linkage to treatment Yes No

Incidence of social harms Yes No

**Part 3:RESULTS**

**Sample size:**

**Missing participants(numbers):**

**Reasons for missing participants:**

**Missing data to be repoteted from the author:**

[any communication with the author(s)?] Yes No

**If yes,please specify below:**

**Measure of Outcomes Total numbers (%)**

**Uptake of HIV testing:** (Number or % of individuals who underwent HIV testing and counselling (HTC) and received their test results over those who were offered HTC)

**Yield of new diagnosis** (Number or % of individuals who were newly-diagnosed HIV–positive over those who were offered HTC)

**Prevalence of newly diagnosed HIV positive:** (number or % of individuals who were newly diagnosed HIV–positive over those who underwent HTC)

**Linkage to HIV prevention:**

- Voluntary medical male circumcision Yes No
- Visit family planning clinic Yes No

**Linkage to care:**

Criteria for care: CD4 ≤, 200cell/ µl : CD4 201-350 cell/µl CD4 ≥ 351 cells/ µl

**Linkage to treatment:**

Enrolled in ART using WHO criteria (CD4 ≤ 350) Yes No

**Social harms:** (Number or % of participants who report any episode of harm during or after HIV testing)

**Measure of uptake Measure of prevalence**

Crude measure Crude measure

Adjusted measure Adjusted measure

**Measure of yield Measure of social harm**

Crude measure Crude measure

Adjusted measure Adjusted measure

If **adjusted** what factors were adjusted for in this study (list):

**List of social harms**

**1.**

**2.**

**3.**

**4.**

**Risk of bias assessment for experimental studies** (see attachment for risk of bias assessment)

| **Sequence generation** | |
| --- | --- |
| State reasons below for grading | Grade(circle) |
|  | Adequate |
|  | Inadequate |
|  | Unclear |
| **Concealment of allocation** | |
| State reasons below for grading | Grade(circle) |
|  | Adequate |
|  | Inadequate |
|  | Unclear |
| **Blinding/masking** | |
| State reasons below for grading | Grade(circle) |
|  | Adequate |
|  | Inadequate |
|  | Unclear |
| **Incomplete outcome data** | |
| State reasons below for grading | Grade(circle) |
|  | Adequate |
|  | Inadequate |
|  | Unclear |
| **Selective outcome reporting** | |
| State reasons below for grading | Grade(circle) |
|  | Adequate |
|  | Inadequate |
|  | Unclear |

**Quality assessment** (observational studies)

**A: NEWCASTLE - OTTAWA QUALITY ASSESSMENT SCALE CASE CONTROL**

**STUDIES**

Note: A study can be awarded a maximum of one star for each numbered item within the Selection

and Exposure categories. A maximum of two stars can be given for Comparability.

**Selection**

1) Is the case definition adequate?

a) Yes, with independent validation.

b) Yes, e.g., record linkage or based on self reports.

c) No description.

2) Representativeness of the cases.

a) Consecutive or obviously representative series of cases.

b) Potential for selection biases or not stated.

3) Selection of Controls

a) Community controls.

b) Hospital controls.

c) No description.

4) Definition of Controls.

a) No history of disease (endpoint).

b) No description of source.

**Comparability**

1) Comparability of cases and controls on the basis of the design or analysis.

a) Study controls for _______________ (Select the most important factor).

b) Study controls for any additional factor (This criteria could be modified to indicate specific

control for a second important factor).

**Exposure**

1) Ascertainment of exposure.

a) Secure record (e.g. surgical records).

b) Structured interview where blind to case/control status.

c) Interview not blinded to case/control status.

d) Written self report or medical record only.

e) No description.

2) Same method of ascertainment for cases and controls.

a) Yes.

b) No.

3) Non-Response rate.

a) Same rate for both groups.

b) No respondents described.

c) Rate different and no designation.

**B: NEWCASTLE - OTTAWA QUALITY ASSESSMENT SCALE COHORT STUDIES**

Note: A study can be awarded a maximum of one star for each numbered item within the Selection

and Outcome categories. A maximum of two stars can be given for Comparability.

**Selection**

1) Representativeness of the exposed cohort.

a) Truly representative of the average _______________ (describe) in the community.

b) Somewhat representative of the average ______________ in the community.

c) Selected group of users (e.g. nurses, volunteers).

d) No description of the derivation of the cohort.

2) Selection of the non exposed cohort.

a) Drawn from the same community as the exposed cohort

b) Drawn from a different source

c) No description of the derivation of the non exposed cohort

3) Ascertainment of exposure.

a) Secure record (e.g. surgical records).

b) Structured interview.

c) Written self report.

d) No description.

4) Demonstration that outcome of interest was not present at start of study.

a) Yes.

b) No.

**Comparability**

1) Comparability of cohorts on the basis of the design or analysis.

a) Study controls for_____________ (select the most important factor).

b) Study controls for any additional factor (This criteria could be modified to indicate specific

control for a second important factor).

**Outcome**

1) Assessment of outcome.

a) Independent blind assessment.

b) Record linkage.

c) Self report.

d) No description.

2) Was follow-up long enough for outcomes to occur___

a) Yes (select an adequate follow up period for outcome of interest)

b) No.

3) Adequacy of follow up of cohorts

a) Complete follow up - all subjects accounted for

b) Subjects lost to follow up unlikely to introduce bias - small number lost - > ____ % (select an adequate %), follow up, or description provided of those lost).

c) Follow up rate < ____% (select an adequate %) and no description of those lost.

d) No statement.
